# Accurate Spectral Properties within Double-Hybrid Density Functional Theory: A Spin-Scaled Range-Separated Second-Order Algebraic-Diagrammatic Construction-Based Approach

**DOI:** 10.1021/acs.jctc.1c01100

**Published:** 2022-01-13

**Authors:** Dávid Mester, Mihály Kállay

**Affiliations:** Department of Physical Chemistry and Materials Science, Budapest University of Technology and Economics, P.O. Box 91, H-1521 Budapest, Hungary

## Abstract

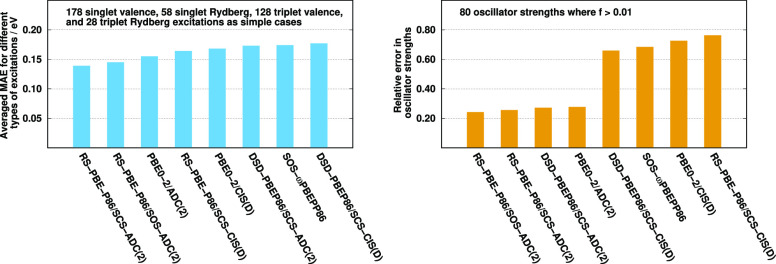

Our second-order algebraic-diagrammatic construction [ADC(2)]-based double-hybrid (DH) ansatz (*J. Chem. Theory Comput.***2019**, *15*, 4440. DOI: 10.1021/acs.jctc.9b00391) is combined with range-separation techniques. In the present scheme, both the exchange and the correlation contributions are range-separated, while spin-scaling approaches are also applied. The new methods are thoroughly tested for the most popular benchmark sets including 250 singlet and 156 triplet excitations, as well as 80 oscillator strengths. It is demonstrated that the range separation for the correlation contributions is highly recommended for both the genuine and the ADC(2)-based DH approaches. Our results show that the latter scheme slightly but consistently outperforms the former one for single excitation dominated transitions. Furthermore, states with larger fractions of double excitations are assessed as well, and challenging charge-transfer excitations are also discussed, where the recently proposed spin-scaled long-range corrected DHs fail. The suggested iterative fourth-power scaling RS-PBE-P86/SOS-ADC(2) method, using only three adjustable parameters, provides the most robust and accurate excitation energies within the DH theory. In addition, the relative error of the oscillator strengths is reduced by 65% compared to the best genuine DH functionals.

## 1 Introduction

Nowadays, density functional theory (DFT) is one of the most popular tools in quantum chemistry, which offers an appropriate compromise between accuracy and computational time. The performance of the functionals for different ground-state properties is well-known through comprehensive benchmark studies;^1−5^ however, their applicability in a black box manner is often in question. Accordingly, one of the most essential requirements from the community is the development of robust approaches for general applications.

To investigate time-dependent properties of molecular systems, such as excitation energies, oscillator strengths, polarizabilities, and chiroptical properties, time-dependent DFT (TDFFT) is the most common choice.^6−11^ It can be derived from DFT through the linear-response formalism, and similar to ground-state calculations, its computational demands are fairly low. However, the formally exact theory suffers from the same drawbacks as the ground-state analogue. That is, the wrong long-range (LR) behavior of the exchange-correlation (XC) functionals is well-known, which causes significant problems for weak interactions, Rydberg and charge transfer (CT) states, or π → π* excitations of conjugated systems.^12−16^ Consequently, adequate results cannot be expected from TDDFT using pure XC functionals. For semiquantitative accuracy, at least hybrid functionals are recommended, where the XC energy contains a Hartree–Fock (HF) exchange contribution as well. This inclusion improves the results; however, hybrid functionals can still fail for challenging cases, and their general usage requires further developments.

To remedy the wrong LR behavior, a range-separated (RS) scheme was proposed by Savin and co-workers^17,18^ where the Coulomb operator is split into LR and short-range (SR) components. For hybrid functionals relying on this approach,^19−25^ the LR (SR) part of the exchange energy is dominantly covered by the LR HF (SR DFT) energy, while the DFT correlation contribution is left unaltered. The improvements over the standard hybrids have been demonstrated in excellent studies.^1,2,26−29^ Besides the aforementioned problem, only states dominated by one-electron excitations can be modeled within TDDFT. To cure this problem, an alternative choice can be the so-called dressed TDDFT formalism.^30−33^ For such approaches, the explicit inclusion of double and higher excitations was elaborated which enables the better description of transitions with larger fractions of double excitations.^34−36^

The performance of density functional approximations can also be improved by combining them with wave function methods. In the case of double-hybrid (DH) approaches,^37^ a hybrid Kohn–Sham (KS) calculation is carried out, and a second-order Møller–Plesset (MP2)-like correction evaluated on the KS orbitals is added to the XC energy. The parametrization of the first DHs were based on empirical considerations,^37−39^ while nonempirical approaches^40−45^ were later derived from the adiabatic connection formalism. As it was pointed out later, functionals using empirical parametrization are more suitable for ground-state applications.^3^ Spin-scaled DH variants^46−54^ were also proposed, where the MP2 contribution is replaced by the spin-component-scaled (SCS)^55^ or scaled-opposite-spin (SOS)^56^ MP2 correction. The DH approximation was extended to excited states by Grimme and Neese.^57^ In their approach, a hybrid TDDFT calculation is performed, and subsequently, the second-order contribution is added *a posteriori* relying on the configuration interaction singles (CIS)^58^ with perturbative second-order correction [CIS(D)]^59^ method. Later, several DH functionals were adapted to excited-state calculations,^60,61^ and the most encouraging ones were also combined with spin-scaling techniques.^62,63^ The accuracy and efficiency of DH functionals have been demonstrated in numerous studies, and their superiority to conventional DFT methods has been proven.^1,2,4,5,54,64−67^

Besides CIS(D), the second-order algebraic-diagrammatic construction [ADC(2)] method^68^ can also be considered as a natural excited-state extension of the MP2 method. It was elaborated through the diagrammatic perturbation expansion of the polarization propagator and the Møller–Plesset partitioning of the Hamiltonian. Over the past decade, the scope of ADC(2) has been significantly extended by Dreuw and co-workers,^69−75^ Köhn and co-workers,^76,77^ and Hättig and Winter.^78,79^ Recently, we have shown that an excited-state DH analogue can also be defined relying on it.^80^ We have also demonstrated that the ADC(2)-based DHs outperform the CIS(D)-based ones, especially for excited states with larger fractions of double excitations and transition strengths.

The RS and DH approaches can also be utilized together. The first attempts in this direction were made by Ángyán and co-workers,^81,82^ while the necessary technicalities were elaborated by Toulouse et al.^83,84^ and Stoll and co-workers.^85−87^ Inspired by these studies, several RS-DH approaches were proposed for ground-state^53,88^ and excited-state calculations^89,90^ as well. The more approximate form of the theory, the family of the so-called long-range corrected (LC) functionals, is also noteworthy where solely the exchange contributions are range-separated.^46,91−94^ For such functionals, an excited-state analogue was recently proposed by Goerigk and co-workers.^95−98^

In this paper, we combine our ADC(2)-based DH ansatz^80^ with range-separation techniques. First, we give a brief overview of the corresponding theories. Thereafter, we assess the different XC kernels and compare the standard, LC-DH, and RS-DH functionals. In this section, the role of range separation is emphasized. Finally, we demonstrate the robustness of our ansatz through numerous benchmark calculations using only high-quality reference values. These excitation energies and oscillator strengths were calculated at the coupled-cluster (CC) level including triple excitation corrections, such as the CC3,^99^ CCSDR(3),^100^ and CCSDT-3^101^ approaches. We note that several terms are used in this study with similar meanings. Accordingly, to help the reader, these terms are used consistently. In the case of *genuine* functionals, the CIS(D)-based approach is referred to. For *standard* functionals, no range separation is invoked for the XC energy. These functionals can also be named the global ones. If the method is *original*, no spin-scaling techniques are applied to the correlation contributions.

## 2 Theory and Methodology

### 2.1 Energy Expressions in Genuine DH Theory

In the very first approach of the standard DH theory,^37^ the ground-state XC energy is expressed as
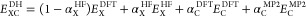
1where *E*_X_^DFT^ and *E*_X_^HF^ denote the semilocal DFT and exact HF exchange energies, respectively, and *E*_C_^DFT^ stands for the DFT correlation energy contribution, while *E*_C_^MP2^ is the MP2 correlation energy. The expression contains three adjustable parameters as the ratio of the HF and DFT contributions to the exchange energy is handled by a single mixing factor α_X_^HF^, while the DFT and MP2 correlations are scaled by the coefficients α_C_^DFT^ and α_C_^MP2^, respectively. In general, although not exclusively, the α_C_^DFT^ + α_C_^MP2^ = 1 condition is invoked which reduces the number of the independent parameters by one. Numerous standard DH functionals with empirical^37−39^ and nonempirical^42−45^ parametrization were elaborated in the past few years.

Later, a simple one-parameter double-hybrid approximation was proposed by Savin et al.,^40^ where the ground-state XC energy is obtained as
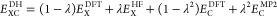
2

In this approach, the ansatz contains only one adjustable parameter, λ, which can be interpreted as the weight of the wave function methods in the XC energy. The above equation exactly corresponds to the most commonly used form of eq 1 with parameters λ = α_X_^HF^ and λ^2^ = α_C_^DFT^.

The DH results can be further improved via range-separation techniques. One of the simplest attempts is the long-range correction.^19^ In the flavor of DHs,^46,91^ solely the exchange contributions are range-separated, while the correlation part is retained. For such functionals, the XC energy is defined by
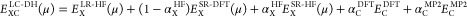
3where the amounts of the SR DFT and HF exchange contributions, denoted by *E*_X_^SR-DFT^ and *E*_X_^SR-HF^, are controlled by the mixing factor α_X_^HF^, and the total LR HF exchange energy contribution, *E*_X_^LR-HF^, is added to the XC energy. The range-separation parameter μ controls the transition between the SR and LR parts. A similar LC expression can be put for eq 2 as
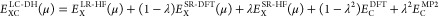
4To the best of our knowledge, the kernel of the XC functional shown in the above expression has not been studied in the literature so far. As it can be seen, these LC formulas contain one more adjustable parameter compared to eqs 1 and 2, and the corresponding standard DH expressions are recovered in the μ = 0 limit; however, no other ansatz is retrieved in the μ → *∞* limit.

A more elaborate ansatz was proposed by Toulouse et al.^88^ In their two-parameter approach, both the exchange and the correlation contributions are range-separated. For such RS DHs, the XC energy is obtained as
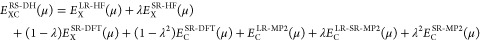
5where *E*_C_^SR-MP2^ and *E*_C_^SR-DFT^ stand for the SR MP2 and DFT correlation contributions, respectively, and *E*_C_^LR-MP2^ is the LR MP2 energy, while *E*_C_^LR-SR-MP2^ denotes the mixed LR-SR contribution. This expression contains only two adjustable parameters similar to eq 4, which means that the effects of the range separation for the correlation part can be easily assessed. We also note that well-defined energy formulas are retrieved in both limits of parameter μ. First, eq 2 is recovered for μ = 0, while in the μ → *∞* limit, the approach simplifies to the standard MP2 method. The effective implementation of such RS DHs, the corresponding working equations, and the calculation of the *E*_C_^SR-DFT^ contribution were previously discussed in detail in ref (89).

The genuine DH calculations are carried out in a two-step manner.^37^ First, the self-consistent hybrid KS equations are solved including the corresponding HF exchange contributions, as well as the DFT exchange and correlation potentials. Thereafter, the XC energy is augmented with an MP2-like correction evaluated on the KS orbitals obtained. Note that other schemes, i.e., the so-called xDH variants,^52,65,102,103^ also exist; however, the application of such functionals for excited-state calculations has not been elaborated. For all the aforementioned energy expressions, spin-scaled variants can also be defined,^46−52,54,90^ where the perturbative correction is replaced by the SCS^55^ or SOS^56^ MP2 energy. In this case, the opposite-spin (OS) and same-spin (SS) contributions to the MP2 correlation energy are scaled separately, which enables higher flexibility of the energy functional; however, the number of empirical parameters increases at the same time. The scaling factors of the OS and SS contributions are denoted by α_C_^OS^ and α_C_^SS^, respectively. The computational scaling of the SOS variant can be reduced to *N*^4^ invoking the density fitting approximation for the electron-repulsion integrals and Laplace transform-based techniques, whereas the scaling of the original and SCS variants are *N*^5^, where *N* is a measure of the system size.

In the most common extension of DH theory for excited-state calculations,^57^ the excitation energy is also obtained in two steps. First, a Hermitian eigenvalue equation relying on the Tamm–Dancoff approximation (TDA)^104^ is solved as
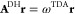
6where **A**^DH^ denotes the corresponding Jacobian, **r** is the singles excitation vector, and ω^TDA^ stands for the TDA excitation energy. As the Jacobian contains the second derivative of the XC energy, its matrix elements depend on which expression is used of eqs 1–5. Note that, as it was also presented in ref (57), the excitation energy and the singles excitation vector can be obtained from the full TDDFT^6^ equations as well. Reliable singlet excitation energies can be attained within this theory;^62,96^ however, it is not recommended for general applications because of the triplet instability of TDDFT.^10,97,105^ Having the TDA solution at hand, the second-order correction is calculated perturbatively relying on the CIS(D)^59^ method. If the range separation is not applied to the correlation part, the final excitation energy is calculated as^57,95^
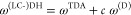
7where ω^(D)^ stands for the second-order correction, and *c* is a scaling factor. This factor is equal to α_C_^MP2^ in the case of eq 1 or 3, while *c* = λ^2^ for the one-parameter DHs and their LC variant (see eqs 2 and 4). For the more elaborate RS DHs, the final excitation energy is proposed to be evaluated as^89^
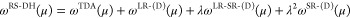
8where ω^LR-(D)^, ω^SR-(D)^, and ω^LR-SR-(D)^ denote the LR, SR, and mixed contributions to the perturbative correction, respectively. Note that the TDA solution, and thus the second-order correction, depends on the range-separation parameter in the case of LC DHs as well; however, for the sake of simplicity, this notation is omitted in eq 7. Spin-scaled variants can also be defined for excited-state DH calculations,^62,90,98^ relying on the SCS-CIS(D) method.^63,106,107^ As three various parametrizations of SCS-CIS(D) exist, we briefly discuss the differences. The authors in ref (63) scaled the SS and OS contributions by different parameters in the “direct” and “indirect” terms of the CIS(D) correction resulting in four adjustable parameters. In contrast, Grimme et al.^106^ scaled only the indirect terms with two empirical factors regarding the SS and OS contributions. In both cases, the adjustable parameters were tuned for excitation energies. In addition, a spin-scaled ADC(2)-consistent analogue was proposed by Hättig et al.,^107^ where the same mixing factors were used for both terms retained from the ground-state theory. As it was pointed out in ref (67), the approach of Rhee and Head-Gordon^63^ is superior; however, this, at least partly, can be explained by the higher level of parametrization. In this work, we follow the approach of ref (107), which can be justified by three arguments. First, it is advantageous to keep the number of empirical parameters as low as possible. Second, the main scope of this paper is to compare the genuine and ADC(2)-based DHs. As this approach is consistent with the spin-scaled ADC(2)^78,107^ theory, it forces us to use this approach. Finally, we would like to retain the consistency with our previous works.^80,90^

### 2.2 ADC(2) Theory

In ADC(2) theory,^68,108−110^ the ground-state ADC(2) correlation energy is simply approximated by the MP2 energy, while the first-order ground-state wave function is defined by
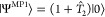
9where |0⟩ is the HF determinant, and cluster operator
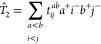
10generates cluster amplitudes *t*_*ij*_^*ab*^ associated with the *a*^+^ and *i*^–^ creation and annihilation operators, respectively, acting on the corresponding spin orbitals. Here, *a* and *b* (*i* and *j*) refer to virtual (occupied) orbitals, whereas *p* and *q* denote generic orbitals. For convenience, the  shorthand notation is introduced, where *n* stands for *n*-fold excitation.

The ADC(2) ansatz for the wave function of the excited states is given in the form of
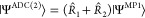
11where the spin-coupled single and double excitation operators, *R̂*_1_ and *R̂*_2_, can be defined similar to eq 10. The excitation energy, being correct up to second order, can be obtained via the diagonalization of the following Hermitian Jacobian^68,109^
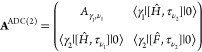
12where *Ĥ* is the Hamiltonian, *F̂* denotes the Fockian, and |γ_*n*_⟩ stands for *n*-fold excited determinants. The elements of the singles–singles block can be expressed as , where the CIS Jacobian is defined by
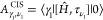
13while the second-order contributions to the singles–singles block are calculated as
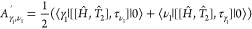
14

In practice, the problem is recast as a nonlinear eigenvalue equation
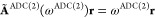
15where ω^ADC(2)^ is the ADC(2) excitation energy. The benefit is that the resulting equation with the effective Jacobian matrix  has to be solved only for the single excitation coefficients, while the doubles amplitudes can be calculated on the fly, and their storage can be avoided.^111^ The elements of the effective Jacobian read explicitly as
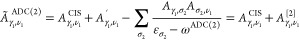
16where  stands for the difference of the orbital energies, and the terms including the second-order contributions are collected into matrix **A**^[2]^. At the end of the iterative procedure, the converged ADC(2) solution vector is normalized, and the transition density matrix required for the ground- to excited-state transition moments is computed as
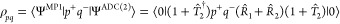
17

This expression is often simplified^76,80,112^ by discarding disconnected contributions and by neglecting the higher than fifth-power-scaling second-order terms. It can be shown that, analogously to the approximate coupled-cluster singles and doubles method CC2, the resulting ADC(2) density matrix is consistent with the linear-response CC theory and correct up to first order.

### 2.3 ADC(2)-Based DH Theory

As ADC(2) can also be regarded as one of the natural excited-state extensions of the MP2 method; similar to the CIS(D) approach in Section 2.1, an ADC(2)-based DH analogue can be proposed as well. In our previous work,^80^ a combined DH-ADC(2) scheme has been introduced for standard DHs. In that case, an ADC(2)-like calculation is performed with a modified effective Jacobian , where **A**^CIS^ is replaced by **A**^DH^ derived from eq 1 or 2, and the second-order terms are scaled by an empirical factor. That is, the modified matrix read as
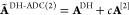
18where, of course, *c* = α_C_^MP2^ in the case of eq 1, while *c* = λ^2^ for one-parameter DHs (see eq 2). Here, we combine this ansatz with range separation.

Concerning the LC DH variants, similar expressions can be obtained. In such cases, the Jacobian **A**^DH^ is defined according to eq 3 or 4, while the standard second-order term is scaled by the corresponding factor. Note that, due to the range separation in the exchange part, this ansatz contains one more adjustable parameter compared to the standard DH-ADC(2) expressions. The spectral intensities for both the original DH-ADC(2) model and its LC variant can be calculated with a minor modification to eq 17. That is, the contribution linear in *R̂*_1_ is separated, and the remaining terms are scaled by the empirical factor of the second-order terms
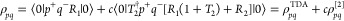
19

As the range separation can be applied to the correlation contributions in the ADC(2) theory, a similar approach can also be introduced for the more elaborate RS DHs as well. In that case, the equations are somewhat different. Analogously to eq 8, the corresponding second-order contributions have to be calculated separately; thus, the expression for the effective Jacobian reads as
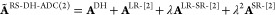
20while the transition density matrix is obtained as
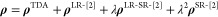
21using the corresponding range-separated *R̂*_2_ coefficients and *T̂*_2_ amplitudes.

All the aforementioned ADC(2)-based approaches using any XC kernel contain the same number of empirical parameters as their CIS(D)-based counterparts, and the same statements hold for the limits of parameter μ as in Section 2.1. That is, for the LC DHs, the corresponding standard DH expressions are recovered in the μ = 0 limit; however, no other ansatz is retrieved if μ → *∞*. In contrast, for the RS DHs, the standard one-parameter DH excitation energy is recovered for μ = 0, while in the μ → *∞* limit, the approach simplifies to the original ADC(2) method. Inspecting the mixing factors, α_X_^HF^, α_C_^DFT^, α_C_^MP2^, or λ, and their limits, this transferability between the CIS(D)- and ADC(2)-based approaches exists as well. In addition, spin-scaled variants can also be defined for the ADC(2)-based DHs.^80^ In that case, just as for the SCS^107^ and SOS^78^ variants of ADC(2), the OS and SS contributions in the corresponding **A**^[2]^ and **ρ**^[2]^ matrices are scaled separately. It is important to note that the computational scaling of SOS-ADC(2) is still *N*^4^ similar to the SOS-CIS(D) correction, but the procedure is iterative. The computational cost of a single iteration in the proposed ADC(2)-based RS-DH approaches is practically identical to the time required for the CIS(D) correction in the case of the genuine RS-DH functionals. To be fair, we note that these second-order corrections are more demanding as the full-, short-, and long-range contributions are also required (see eqs 8 and 20); however, the scaling of the procedure does not change. The corresponding timings for the genuine RS-DH functionals were presented in detail in ref (89). In addition, the computational cost of the standard ADC(2)-based approaches was also discussed in ref (80), in comparison with the standard genuine functionals.

The benefits of the standard ADC(2)-based approach compared to the genuine DH methods were discussed in detail in ref (80). Accordingly, we now focus only on the most significant differences. First, in the case of CIS(D)-based approaches, the doubles correction is added *a posteriori* to the TDA excitation energy, while these excitations are treated iteratively in the new ansatz. Thus, concerning excitation energies, the ADC(2)-based approaches moderately but consistently outperform the genuine DH methods; furthermore, this improvement is especially noticeable when the weights of double excitations are relatively large in the excited-state wave function. Second, as the perturbative correction is only an energy correction for the CIS(D)-based DH approaches, the oscillator strengths have just hybrid quality. In contrast, the new methods also allow us to evaluate the transition moments at a higher level taking into account the effect of double excitations, which considerably raises the quality of the computed oscillator strengths.

## 3 Results

In what follows, we demonstrate the advantages of the ADC(2)-based ansatze over the genuine DH approach, regardless of which XC kernel is chosen. This has been carried out, at least partly, for standard DHs in ref (80), which is extended here to the RS variants. The necessity of the range separation for CT excitations has been demonstrated in several papers.^89,90,95,97,98,113,114^ Accordingly, we expect that the robustness of the standard DH-ADC(2) scheme is improved via range separation. In addition, we would like to prove that the range separation for the correlation contributions is highly recommended for both the CIS(D)- and the ADC(2)-based schemes. For this purpose, we compare the performance of different types of functionals using the same number of adjustable parameters.

### 3.1 Computational Details

The new approaches have been implemented in the Mrcc suite of quantum chemical programs and will be available in the next release of the package.^115,116^

For the calculations, Dunning’s correlation consistent basis sets (cc-pV*X*Z, where X = D and T),^117,118^ and their diffuse function augmented variants (aug-cc-pV*X*Z),^119^ and Ahlrichs’ TZVP^120^ basis sets were used. In all calculations, the density-fitting approximation was utilized for both the ground and the excited states, and the corresponding auxiliary bases of Weigend and co-workers^121−123^ were employed. To help the reader, at all the figures or tables the corresponding basis sets are specified. The frozen core approximation was utilized in all the post-KS/HF steps, while the oscillator strengths, denoted by *f*, were computed in the dipole length approximation.

In this study, the exchange and correlation functionals of Perdew, Burke, and Ernzerhof (PBE)^124^ and Perdew’s 1986 correlation functional (P86)^125^ were used. In eq 5, to obtain the SR DFT contributions utilizing the local-scaling approximation,^89,90,126^ the Slater–Dirac exchange^127−129^ and the Perdew–Wang 1992 correlation^130^ functionals were applied as local-density approximation functionals together with their SR extensions proposed by Savin^131^ and Paziani et al.^132^ The built-in functionals of the Mrcc package were used in all cases, except for ωPBEPP86 and its spin-scaled variant^98^ where the locally modified version of the Libxc library^133,134^ was employed.

In order to retain the consistency with the previous DH studies,^62,80,89,90,95,97,98^ our training and benchmark sets were selected from the literature. For most of them, high-quality singlet and triplet excitation energies are also available, while two compilations provide oscillator strengths as well. The adjustable parameters were optimized on the singlet excitations of the well-balanced benchmark set of Gordon et al,^135^ including 32 valence and 31 Rydberg excitations for 14 organic molecules. For this test set, the reoptimized geometries and the composite CC3-CCSDR(3)/aug-cc-pVTZ reference excitation energies of Schwabe and Goerigk^62^ were taken. The updated triplet transitions were recently published by Casanova-Páez and Goerigk,^97^ but this compilation is somewhat less balanced and contains 28 valence and 10 Rydberg excitations obtained at the same level as the singlet ones.

Cross-validation has been performed on several popular benchmark sets. The test set of Thiel and co-workers^136,137^ is a compilation of CC3 excitation energies and oscillator strengths within the linear-response formalism obtained with the TZVP basis set. This test set only incorporates valence excitations, and 121 singlet and 71 triplet excitations of 24 molecules were selected. The singlet transitions were later reconsidered by Kánnár and Szalay,^138^ and these results were used as reference in this study. It is important to note that this compilation contains a relatively large amount of excitations where the weights of double excitations is significant. The first benchmark set^139^ from the QUEST database^140^ proposed by Loos, Jacquemin, and co-workers is also assessed. This compilation, which is hereafter referred to as the LJ1 set, contains 52 singlet (27 Rydberg and 25 valence) and 47 triplet (18 Rydberg and 29 valence) “safe” values of small organic molecules, and CC3/aug-cc-pVTZ excitation energies were considered as reference. The benchmark set contains oscillator strengths within the linear-response formalism obtained at the same level as well. Finally, the challenging intermolecular CT benchmark set recently proposed by Szalay et al.^141^ is also inspected. This set comprises 14 excitation energies evaluated at the CCSDT-3 level using the cc-pVDZ basis set for eight molecular complexes at a large distance to ensure the high CT character of the transitions. All in all, 250 singlet and 156 triplet excitations are involved in this study; furthermore, 80 oscillator strengths are also assessed where *f* > 0.01. We note that, concerning the overall performances, an additional comparison is also carried out where only the unique molecules are considered.

For the excitations energies, the main statistical error measures presented in the tables and figures are the mean error (ME), the mean absolute error (MAE), and the maximum absolute error (MAX). For the oscillator strengths, the MAEs and the relative errors are discussed in detail. All the computed excitation energies, oscillator strengths, and statistical error measures are available in the Supporting Information (SI). In addition, further measures, such as the root-mean-square error (RMSE), standard deviation (SD), and deviation span are also included. These numbers are only discussed if the order of the methods significantly changes when evaluating their performance using the latter measures instead of the former ones.

### 3.2 Determination of the Parameters

First, we compare the quality of the different XC kernels and assess the effects of the range separation for both the LC and RS DHs. For that purpose, we selected different XC parametrizations where the number of the adjustable parameters is two, and thereafter, the empirical parameters were tuned for the same test set. That is, a standard DH using eq 1 is selected where the α_C_^DFT^ + α_C_^MP2^ = 1 condition is invoked, and the two-parameter LC and RS DHs are chosen using eqs 4 and 5, respectively. All the functionals contain the same PBE exchange and P86 correlation functionals and their SR extensions as this is one of the most successful combinations of functionals.^50,89,98,103^ Then, the simultaneous optimization of the parameters was carried out on the singlet excitations of the well-balanced Gordon benchmark set using the aug-cc-pVTZ basis set. The MAE was minimized during the procedure. This fairly objective comparison provides an opportunity for some insight into the quality of the energy expressions as the number of parameters, the training set used, the optimization procedure, and the exchange and correlation functionals included are the same. The study has been carried out for both the genuine and the ADC(2)-based DH approaches. Analogously to our previous works,^89,90^ the standard DH obtained is denoted by PBE-P86, and LC-PBE-P86 stands for the LC variant, while RS-PBE-P86 is the RS-DH approach. For these functionals, at the end of the acronym, it will be labeled whether the genuine or the ADC(2)-based ansatz is used, for example, as PBE-P86/CIS(D) or PBE-P86/ADC(2). The recently proposed LC-DH ωPBEPP86 functional^98^ is also included in this comparison as the adjustable parameters were tuned for the same training set. However, it contains four independent parameters, and the optimization procedure was slightly different than in this study. According to the very comprehensive ranking of ref (98), this functional is considered as the best unscaled LC DH.

For each functional, the expression of the XC energy used and the optimal values obtained during the procedure are collected in Table 1. As it can be seen, the optimal values are practically identical for the genuine and the ADC(2)-based approaches in all the cases. For the standard functionals, the optimal parameters are α_X_^HF^ = 0.68 and α_C_^DFT^ = 0.35. These values are highly in line with the ground-state recommendations as the average percentage of the exact exchange and MP2 correlation are 64% and 32%, respectively, for 50 existing DH functionals.^46^ Inspecting the effects of the long-range correction in the case of ωPBEPP86, the proportion of the HF exchange increases slightly, while that for the MP2 correction is significantly higher compared to the standard DH. The optimal range-separation parameter is 0.18 au, while α_C_^MP2^ + α_C_^DFT^ = 1.16. Interestingly, compared to this functional, almost the same optimal values are obtained for LC-PBE-P86/CIS(D) as λ = α_X_^HF^, λ^2^ ≈ α_C_^MP2^, and μ is identical. We note that the DFT correlation contribution is scaled by 1 – λ^2^ = 0.51 in this case. For the ADC(2)-based variant, λ is unchanged, while μ is negligibly higher. The optimal values for RS-PBE-P86/CIS(D) had been already determined in ref (89), while the same parameters were obtained for the ADC(2)-based approach in this study. In these cases, compared to the two-parameter LC variants, λ is significantly lower, while the range-separation parameter is noticeably higher. These parameters are greatly in line with the ground-state results,^88^ and the trend is also confirmed that the optimal parameter μ is higher when the correlation part is also range-separated^88,126,142−144^ than if only the exchange contributions are.^92,93,95,98^

**Table 1:** XC Kernel Applied, Number of Independent Parameters, and Their Optimal Values Tuned for Singlet Excitations of the Gordon Training Set^62,135^ for Different Functionals Using the aug-cc-pVTZ Basis Set with Corresponding Auxiliary Bases^a^

Functional	XC energy	Number of parameters	α_X_^HF^	α_C_^MP2^	α_C_^DFT^	μ (au)
PBE-P86/CIS(D)	eq 1	2^d^	0.68	0.35	0.65	N/A
PBE-P86/ADC(2)	eq 1	2^d^	0.68	0.35	0.65	N/A
ωPBEPP86^b^	eq 3	4	0.70	0.48	0.68	0.18
LC-PBE-P86/CIS(D)	eq 4	2	0.70	0.49	0.51	0.18
LC-PBE-P86/ADC(2)	eq 4	2	0.70	0.49	0.51	0.19
RS-PBE-P86/CIS(D)^c^	eq 5	2	0.50	0.25	0.75	0.70
RS-PBE-P86/ADC(2)	eq 5	2	0.50	0.25	0.75	0.70

a For the LC- and RS-type functionals, the α_X_^HF^, α_C_^MP2^, and α_C_^DFT^ values correspond, respectively, to λ, λ^2^, and 1 – λ^2^.

b Taken from ref (98).

c Taken from ref (89).

d As α_C_^MP2^ + α_C_^DFT^ = 1.

**Figure 1:**
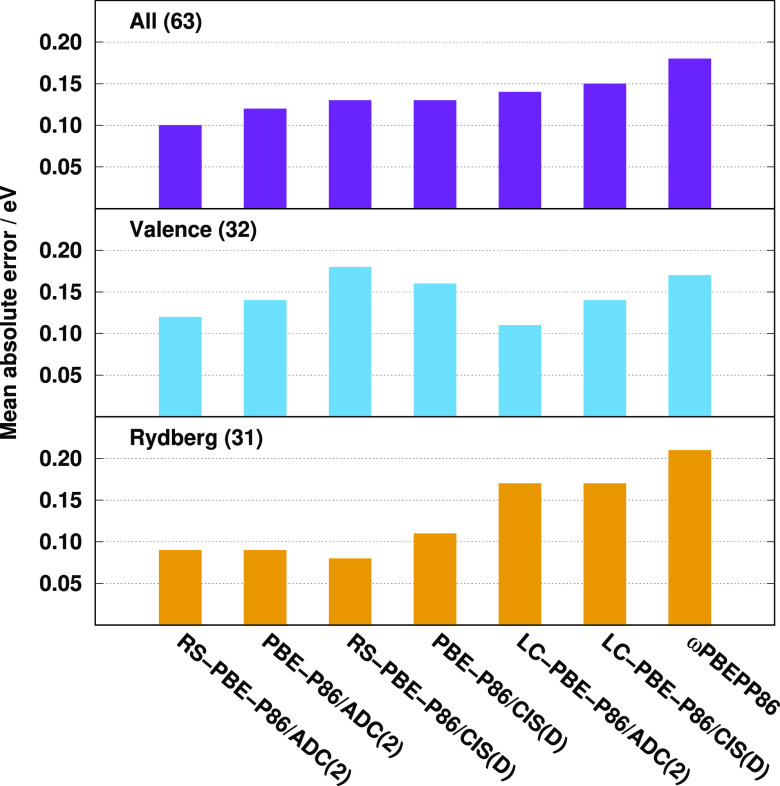
MAEs for singlet excitations of the Gordon training set^62,135^ with optimized parameters for different functionals using aug-cc-pVTZ basis sets with corresponding auxiliary bases. The numbers of transitions are in parentheses.

The MAEs using the default parameters for various types of singlet excitations of the Gordon test set are visualized in Figure 1. Inspecting the bars, several important observations can be made. First, the overall performance of the ADC(2)-based approaches is always better compared to the genuine counterparts. The difference is 0.03 eV for the RS DHs, while it is 0.01 eV for the standard and the LC-DH functionals. The lowest MAEs are attained by the RS DHs; however, the standard DHs outperform the LC variants in both cases. The errors are 0.12 and 0.13 eV for the standard ADC(2)- and CIS(D)-based functionals, respectively. For these methods, the accuracy of the valence excitations is lower compared to the Rydberg results. Interestingly, the long-range correction improves slightly the results on valence excitations; however, the MAEs of the Rydberg values are significantly worse at the same time. In contrast, in the case of the RS DH-ADC(2) approach, the good performance for the valence results is preserved similar to the LC variant, while the Rydberg values are as good as for the standard DHs. For RS-PBE-P86/CIS(D), the MAE of the valence results is somewhat worse; however, the Rydberg values are significantly better compared to the LC analogue. It means that the range separation is highly recommended for both the exchange and correlation terms at the same time.

The most balanced performance is attained by the RS-PBE-P86/ADC(2) approach. The lowest error, 0.10 eV, is also obtained for this functional, while the ωPBEPP86 approach is inferior since its MAE is 0.18 eV. Interestingly, all the functionals contain two adjustable parameters, except for ωPBEPP86, where the number of parameters is four. In addition, three of the four parameters are practically identical to those used for the LC-PBE-P86/CIS(D) approach. In spite of all these, our two-parameter LC scheme provides lower error by 0.03 eV, which can be explained by the facts that the calculation of the SR-DFT exchange contribution and the optimization procedure somewhat differ from what it was carried out in ref (98). As was demonstrated, both the standard and RS approaches outperform the LC variant. In addition, the theoretical background of such functionals was elaborated in refs (97 and^98^), and their performance was discussed in detail in the same papers. Accordingly, hereinafter, further investigation of the LC-PBE-P86 approaches are omitted. In addition, despite the surprisingly good results obtained by the standard DHs, the PBE-P86 functionals are also excluded as their application is out of scope of this study. These standard DHs are only discussed when their failure for intermolecular CT excitations is demonstrated.

**Figure 2:**
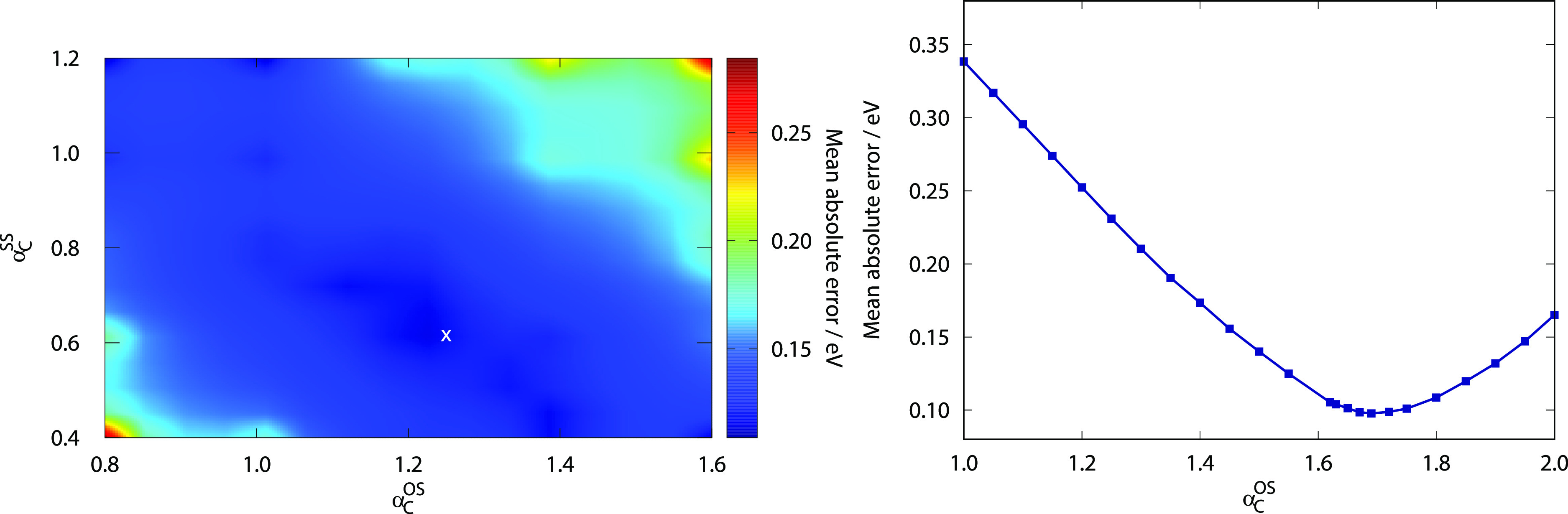
MAEs for singlet excitations of the Gordon training set^62,135^ for SCS (left) and SOS (right) ADC(2)-based variants using aug-cc-pVTZ basis sets with corresponding auxiliary bases. In the case of the SCS variant, the white X marks the global minimum.

Next, we determined the optimal spin-scaling parameters on the same test set using the RS-PBE-P86/ADC(2) functional. For this purpose, the α_C_^OS^ and α_C_^SS^ values were scanned, and the errors for the SCS and SOS variants were minimized. To preserve compatibility with the original approach, the default parameters of λ = 0.5 and μ = 0.7 au were retained. The results are presented in Figure 2. Foremost, we discuss the SCS variant in detail. Unfortunately, as the SCS-ADC(2) problem is iterative, fewer grid points were used during the optimization procedure compared to the CIS(D)-based study in ref (90). However, as it can be seen, the results are highly correlated just as we have seen in the previous paragraphs. That is, concerning the unscaled ansatz as a reference, the MAE slowly decreases with decreasing α_C_^SS^ and increasing α_C_^OS^ parameters. The global minimum can be found at α_C_^OS^ = 1.24 and α_C_^SS^ = 0.64, similar to the genuine ansatz, while the MAE is 0.09 eV at this point. In the case of the SOS variant, the global minimum is well-defined and can be found at α_C_^OS^ = 1.69, again, which corresponds exactly to the CIS(D)-based results. The lowest MAE is 0.10 eV, which is higher only by 0.01 eV compared to the SCS variant. We note that the reoptimization of the parameters, including the λ and μ parameters as well, has only a negligible effect on the results.

### 3.3 Benchmark Calculations

One of the main focuses of this study is to demonstrate the performance of the ADC(2)-based RS DHs on comprehensive benchmark sets. For this purpose, the most successful empirically and nonempirically parametrized standard functionals, namely, DSD-PBEP86^50^ and PBE0-2,^43^ were selected for comparison with our present approaches. On the basis of the available benchmark results for genuine DHs, within the TDA approximation, PBE0-2 outperforms most of the original DHs even with empirical parametrization,^62,90^ while the workhorse spin-scaled variant is DSD-PBEP86.^62^ Some of the deficiencies of these functionals were pointed out in ref (90), such as that the DSD-PBEP86 method has excellent accuracy for valence transitions; however, its error is significantly higher for Rydberg excitations. In contrast, PBE0-2 is somewhat more balanced; that is, it is more accurate for Rydberg excitations; however, its general performance for singlet valence excitations is not outstanding. Furthermore, both functionals failed for challenging intermolecular CT excitations. To be fair, we note that the clear superiority of such standard DHs to global hybrid approaches was demonstrated in several excellent studies.^62,95−97^ We also mention the promising nonempirical PBE-QIDH approach.^45^ Its performance for excited-state calculations was thoroughly benchmarked in refs (89 and 90). As a highly similar accuracy was observed compared to PBE0-2, we omit the detailed discussion of PBE-QIDH. However, we recommend ref (98) for further reading. In this contribution, the outstanding SCS/SOS-PBE-QIDH approaches were introduced, where the spin-scaling factors were tuned for excitation energies. We note that, unfortunately, ADC(2)-based analogues cannot be defined for these functionals as the direct and indirect terms in the second-order correction are scaled separately. In the present comparison, of course, our recently proposed RS-PBE-P86/SCS-CIS(D)^90^ method is also assessed. As it was shown in the original paper, this approach can be considered as one of the most robust and accurate choices for excitation energies within the DH theory. In addition, the SOS variant of the ωPBEPP86 functional, SOS-ωPBEPP86,^98^ is also discussed. This recently proposed functional is considered as the most recommended one from Goerigk’s group; however, other spin-scaled LC-DH functionals are also noteworthy, such as SCS/SOS-ωB88PP86. We note that, in this study, the genuine and the ADC(2)-based variants are also assessed for all the functionals except for SOS-ωPBEPP86. Furthermore, the canonical CIS(D) and ADC(2) results are presented as well. To help the reader, the attributes of the functionals are collected in Table 2.

**Table 2:** Functionals Assessed in Benchmark Calculations^a^

Functional	Exchange	Correlation	Level	Spin scaling	Number of parameters
PBE0-2	PBE	PBE	standard DH	no	2
DSD-PBEP86	PBE	P86	standard DH	yes	4
SOS-ωPBEPP86	PBE	P86	LC DH	yes	5
RS-PBE-P86	PBE	P86	RS DH	yes	3 or 4^b^

a CIS(D)- and ADC(2)-based approaches are discussed for all the functionals except for SOS-ωPBEPP86.

b SOS or SCS variant, respectively.

#### 3.3.1 Gordon Set

**Figure 3:**
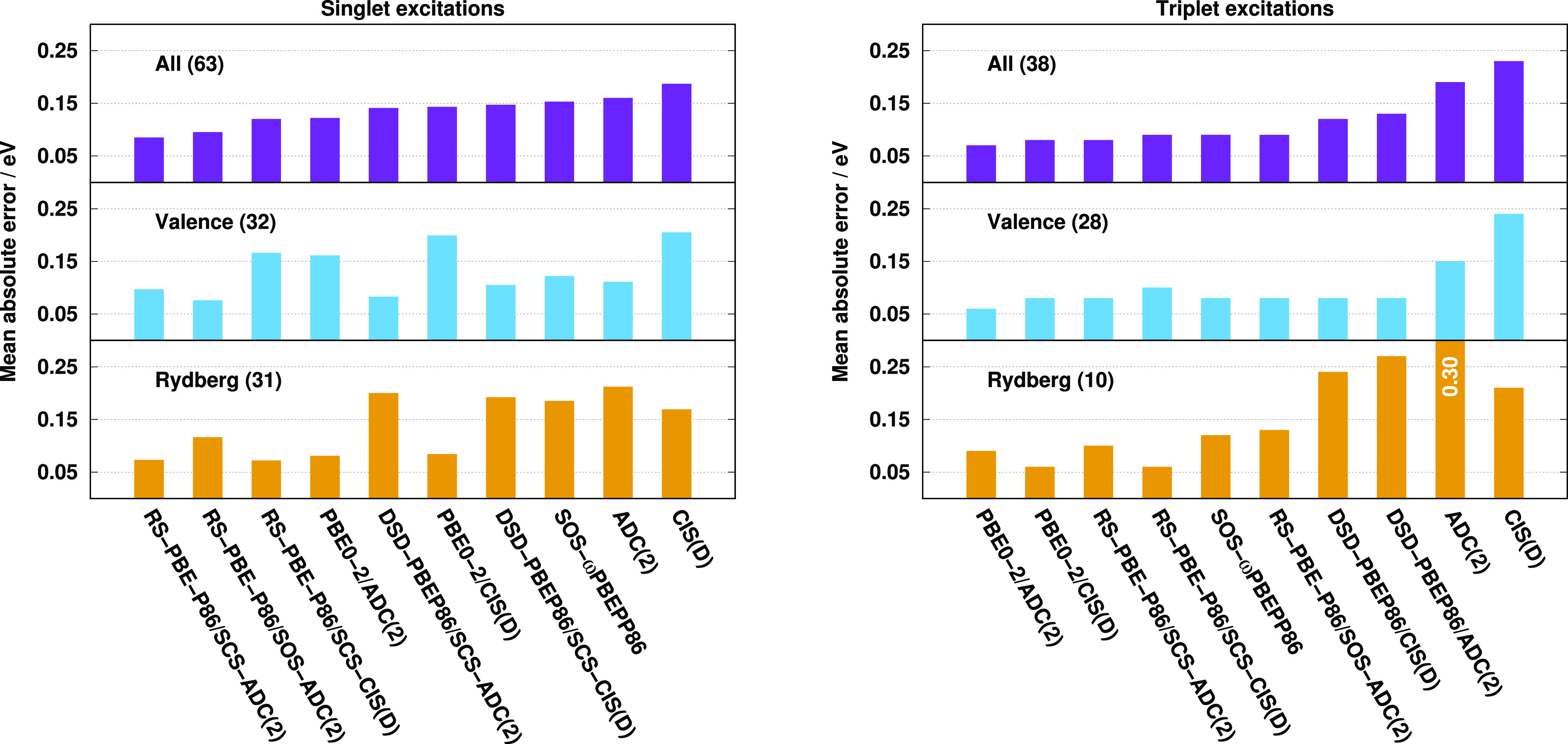
MAEs for calculated singlet (left) and triplet (right) excitation energies for the Gordon test set^97,135^ using aug-cc-pVTZ basis sets with corresponding auxiliary bases. The numbers of transitions are in parentheses.

First, we compare the performances for the Gordon test utilizing the MAEs for the various types of excitations. For an insightful comparison, we note that the adjustable parameters for the RS-PBE-P86 functionals were tuned for the singlet excitations of this test set. In addition, the mixing factors of ωPBEPP86 were also optimized for the same excitations, while the spin-scaling factors of SOS-ωPBEPP86 were tuned for both the singlet and triplet excitations within the same set. The results are visualized in Figure 3. Inspecting the bars for the singlet excitations, we can observe that the best overall performances are attained by the spin-scaled RS DH-ADC(2) approaches. The MAEs are 0.09 and 0.10 eV for the SCS and SOS variants, respectively. The ADC(2)-based methods outperform the CIS(D)-based ones in all the cases. The difference is 0.03 eV for the wave function-based and SCS RS-PBE-P86 approaches, while they are 0.02 and 0.01 eV for the PBE0-2 and DSD-PBEP86 methods, respectively. As the adjustable parameters were trained on this set, the outstanding performance of the RS DHs is not surprising; however, the same set was used for the SOS-ωPBEPP86 as well. Concerning the functionals, this method has one of the largest overall errors with a MAE of 0.15 eV. In the case of valence excitations, the outstanding performance of the DSD-PBEP86 functionals is well-known; nevertheless, significant improvements can be realized for the ADC(2)-based RS DHs compared to the genuine counterpart. The lowest MAEs, 0.08 eV, are achieved by RS-PBE-P86/SOS-ADC(2) and DSD-PBEP86/SCS-ADC(2), while the error does not exceed 0.10 eV for RS-PBE-P86/SCS-ADC(2). Inspecting the Rydberg states, the most outstanding methods are the PBE0-2 approaches and all the RS DHs. In these cases, the error is below 0.08 eV, except for RS-PBE-P86/SOS-ADC(2), where it is still less than 0.12 eV. For the remaining approaches, the MAE is around 0.20 eV. Comparing the ADC(2)-based RS DHs, the SCS variant is noticeably more suitable for Rydberg excitations, while the valence results are somewhat better for the SOS variant.

The MAEs for the triplet excitations are fairly moderate. The overall errors are well-balanced, except for the wave function-based and DSD-PBEP86 approaches, as the largest deviation between the other methods is only 0.02 eV. Again, the ADC(2)-based approaches outperform the genuine variants. The best results are produced by the PBE0-2 functionals, while the RS-PBE-P86/SCS-ADC(2) approach is also outstanding. The MAE is still below 0.10 eV for the other RS and LC DHs. The DSD-PBEP86 functionals are inferior despite the fact that the valence excitations are overrepresented in the test set, while the overall error is even higher for the wave function-based methods. Inspecting the valence results, salient functionals cannot be identified. PBE0-2/ADC(2) is superior with a MAE of 0.06 eV, while the error, precisely 0.10 eV, is still acceptable for RS-PBE-P86/SCS-CIS(D), which is the least favorable case. The MAEs for the Rydberg excitations are less consistent; however, for the best performers, they are not higher compared to the singlet results. The error is 0.06 eV for the genuine PBE0-2/CIS(D) and RS-PBE-P86/SCS-CIS(D) approaches, while it is around 0.10 eV for the ADC(2)-based counterparts. The MAEs for the SOS-ωPBEPP86 and RS-PBE-P86/SOS-ADC(2) methods are still tolerable, while the DSD-PBEP86 functionals are highly not recommended for such excitations. Concerning the valence results, significant differences cannot be observed between the SCS and SOS ADC(2)-based RS DHs, while the SCS variant is somewhat more accurate for Rydberg excitations in this case as well.

The compilation of additional statistical error measures for the Gordon set can be found in Table 3 as well as in the SI. Inspecting the overall MEs for the singlet excitations, the best performances are obtained by the CIS(D)- and ADC(2)-based RS-PBE-P86 methods; however, significant error cancellation between the valence and Rydberg transitions shows up for the former approach. Accordingly, by far the lowest SDs and RMSEs are provided by the SCS and SOS DH-ADC(2) functionals. The SCS variant provides better ME, while the SOS results are somewhat more balanced. A systematic red-shift can be observed between the ADC(2)-based and genuine approaches for the valence excitations, while this effect is less relevant for the Rydberg results. The lowest MAXs, around 0.35 eV, are also attained by the spin-scaled ADC(2)-based RS-PBE-P86 functionals. From this aspect, the PBE0-2/ADC(2) and SOS-ωPBEPP86 methods are also outstanding with a MAX of about 0.40 eV, while it is around 0.60 eV for the others. For the DSD-PBEP86 and wave function-based approaches, the MAX belongs to a Rydberg excitation, while it is affiliated with a valence transition for the others.

**Table 3:** Additional Error Measures for Calculated Excitation Energies (in eV) for the Gordon Test Set^97,135^ Using aug-cc-pVTZ Basis Sets with Corresponding Auxiliary Bases^a^

	Singlet excitations						Triplet excitations					
	All (63)		Valence (32)		Rydberg (31)		All (38)		Valence (28)		Rydberg (10)	
Methods	ME	MAX	ME	MAX	ME	MAX	ME	MAX	ME	MAX	ME	MAX
CIS(D)	–0.03	0.56	0.09	0.54	–0.16	0.56	0.10	0.45	0.20	0.45	–0.19	0.45
ADC(2)	–0.10	0.69	–0.02	0.44	–0.18	0.69	–0.03	0.64	0.06	0.62	–0.30	0.64
SOS-ωPBEPP86	0.10	0.42	0.09	0.42	0.10	0.37	–0.04	0.24	–0.06	0.24	0.03	0.22
RS-PBE-P86/SCS-CIS(D)	0.05	0.62	0.13	0.62	–0.03	0.29	0.02	0.22	0.04	0.22	–0.04	0.14
RS-PBE-P86/SCS-ADC(2)	0.02	0.36	0.07	0.36	–0.03	0.28	–0.04	0.20	–0.03	0.19	–0.08	0.20
RS-PBE-P86/SOS-ADC(2)	0.04	0.35	0.06	0.35	0.02	0.31	0.01	0.19	0.02	0.16	–0.02	0.19
DSD-PBEP86/SCS-CIS(D)	–0.06	0.59	0.08	0.39	–0.19	0.59	–0.07	0.41	–0.01	0.33	–0.24	0.41
DSD-PBEP86/SCS-ADC(2)	–0.08	0.59	0.03	0.23	–0.20	0.59	–0.11	0.45	–0.05	0.37	–0.27	0.45
PBE0-2/CIS(D)	0.11	0.60	0.20	0.60	0.01	0.27	0.02	0.23	0.04	0.23	–0.04	0.14
PBE0-2/ADC(2)	0.08	0.41	0.16	0.41	0.00	0.28	–0.03	0.20	–0.01	0.15	–0.07	0.20

a The numbers of transitions are in parentheses.

In the case of the triplet excitations, the best results are achieved by the RS-PBE-P86/SOS-ADC(2) method with an almost perfect ME. The error is highly acceptable for the others as it is below 0.05 eV, except for the DSD-PBEP86 functionals. Similar findings can be made for the maximum error as well. The lowest MAX, 0.19 eV, is obtained by RS-PBE-P86/SOS-ADC(2), while this measure is only somewhat higher for the remainders, except for the DSD-PBEP86 methods, where the MAXs exceed 0.40 eV. Concerning the reliable functionals, the results are well-balanced, and significant error cancellation between the different types of excitations cannot be observed. The best SDs and RMSEs are provided by the PBE0-2 and RS-DH approaches.

#### 3.3.2 Thiel Set

**Figure 4:**
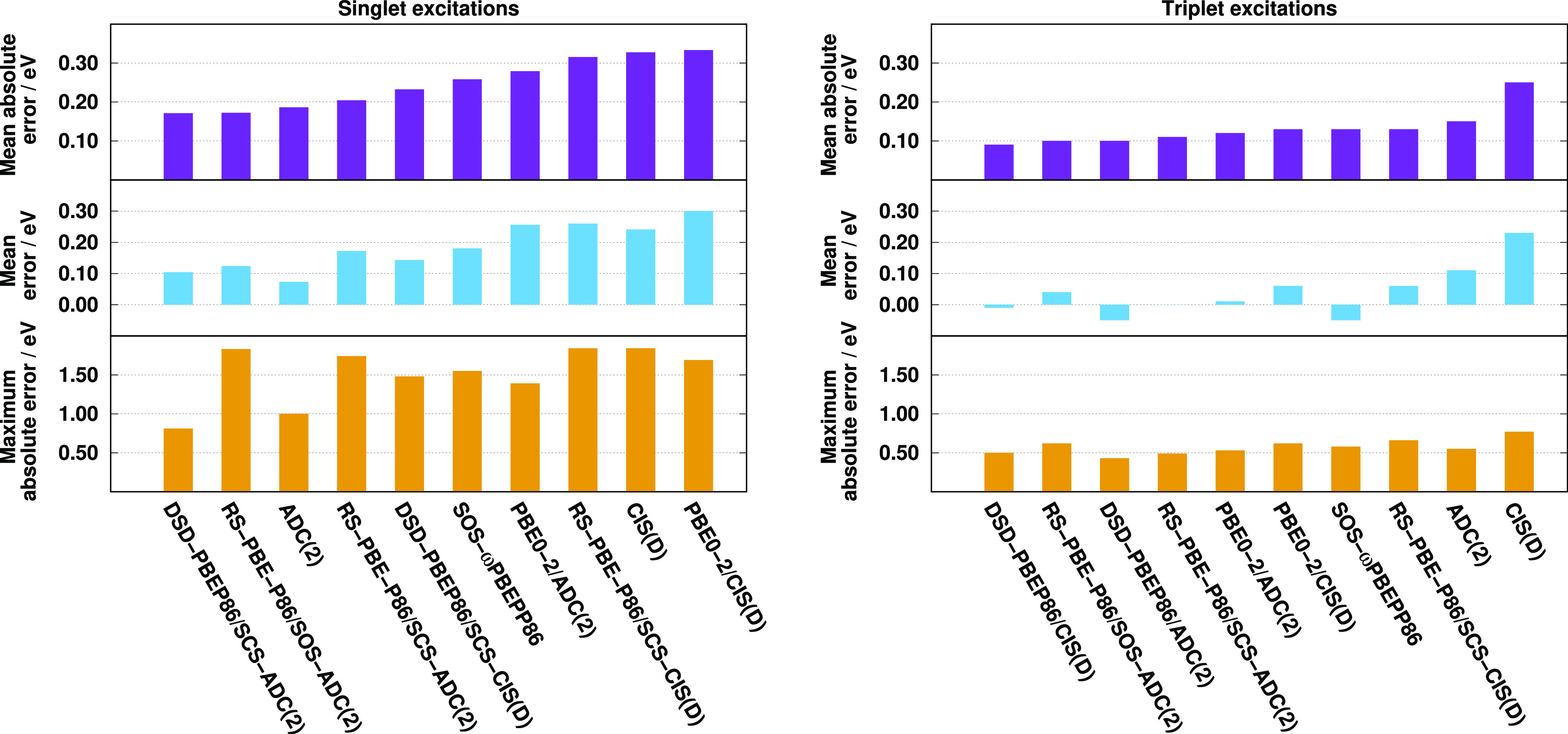
Error measures for calculated singlet (left) and triplet (right) excitation energies for the Thiel test set^136^ using TZVP basis sets with def2-QZVPP-RI(-JK) auxiliary bases. The singlet (triplet) compilation contains 121 (71) transitions.

Next, we assess the methods using the Thiel test set. The obtained error measures are presented in Figure 4. Similar to the singlet valence excitations for the Gordon test set, the best performers are the DSD-PBEP86 and ADC(2)-based RS-DH functionals. The lowest error is achieved by the DSD-PBEP86/SCS-ADC(2) and RS-PBE-P86/SOS-ADC(2) methods with a MAE of 0.17 eV, while it is 0.20 eV for the SCS variant of the latter. As it can be seen, using the ADC(2)-based approach, significant improvements can be gained over the genuine ansatz; however, it is not surprising as ADC(2) has a better performance compared to the CIS(D) approach. The genuine PBE0-2 and SCS RS-PBE-P86 functionals are inferior as the MAEs are higher than 0.30 eV. For all the methods, the excitation energies are systematically overestimated. Outstanding MEs are attained by ADC(2) and DSD-PBEP86/SCS-ADC(2), while they are still acceptable for the best performers. Similar findings can be made for the maximum errors. This measure is also outstanding for the aforementioned methods, while the MAX is fairly well-balanced for the others. Again, the MAEs for the triplet excitations are significantly lower. The same functionals are the most accurate ones with MAEs of around 0.10 eV; however, the order changes somewhat. For the sake of completeness, we mention that the error is also highly acceptable for the remaining functionals as the difference is only 0.04 eV between the best and worst results. Consequently, significant differences cannot be observed between the genuine and ADC(2)-based approaches. At the same time, interestingly, the ADC(2) method is one of the inferiors despite its good performance for the singlet transitions; however, it is still better than CIS(D). The ME and MAX values are also consistent for all the functionals, and salient approaches cannot be identified. The triplet excitation energies are slightly overestimated, except for the DSD-PBEP86 and SOS-ωPBEPP86 approaches, while the MAXs are around 0.50 eV.

**Figure 5:**
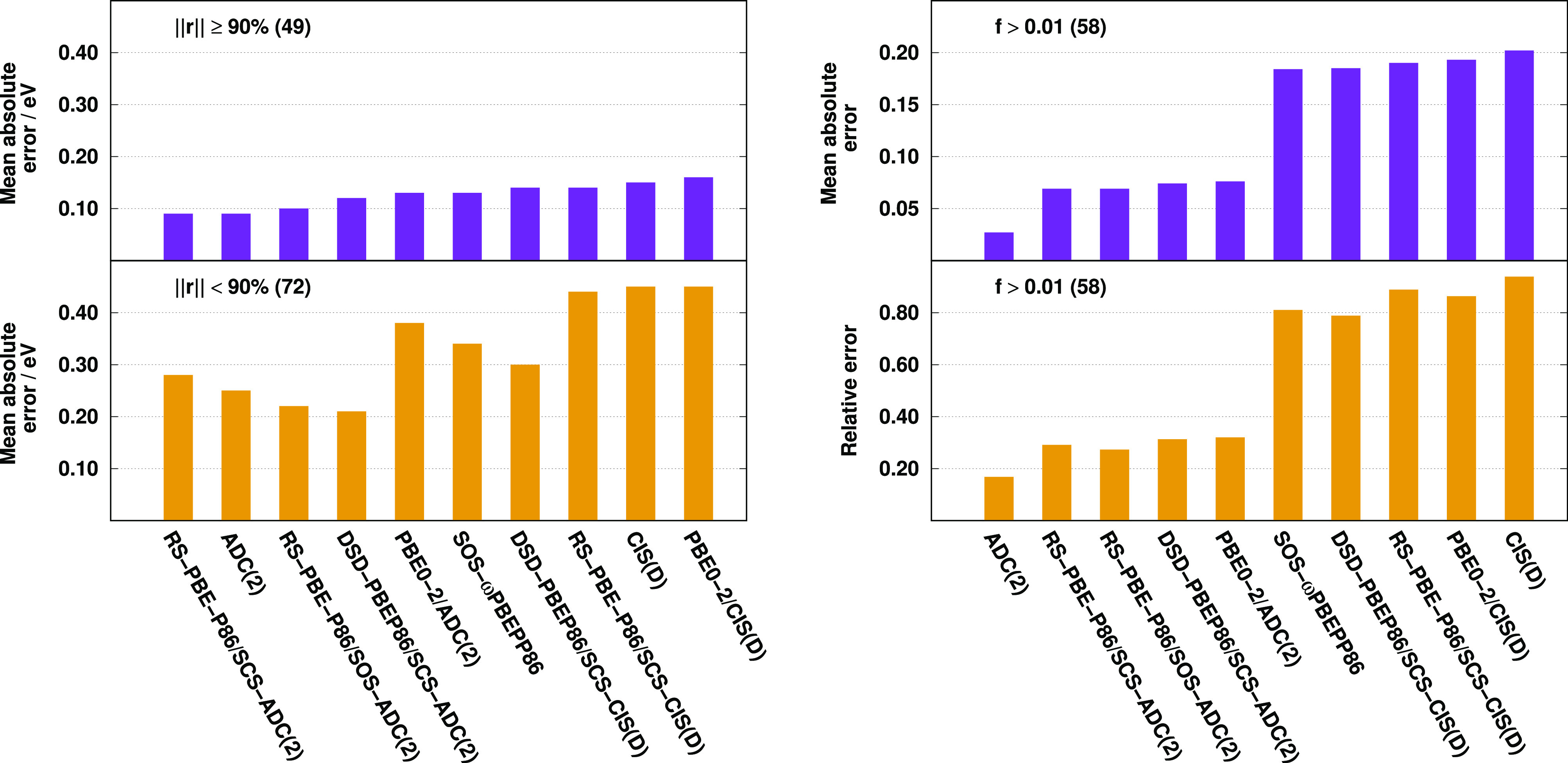
Error measures for calculated singlet excitation energies with different fractions of single excitation coefficients (left) and oscillator strengths (right) for the Thiel test set.^136^ The numbers of transitions are in parentheses.

Presumably, the most significant gains can be realized for transitions with larger fractions of double excitations and oscillator strengths. To assess the first phenomenon, the excitations of the Thiel set were divided into two groups. The first group contains the singles dominated excitations, where the norm of the vector of single excitation coefficients is greater than or equal to 90% in the CC3 wave function, while in the second group, the remaining, transitions with relatively larger fractions of double excitations are included. The error measures for the oscillator strengths are calculated only for states with *f* > 0.01 as small values would bias our results. The results are visualized in Figure 5.

Inspecting the singles dominated excitations, as it can be seen, the results are fairly well-balanced. The ADC(2)-based approaches outperform the CIS(D)-based ones in all the cases; however, the difference is not significant. The improvements are 0.02, 0.03, and 0.05 eV for the DSD-PBEP86, PBE0-2, and SCS RS-DH functionals, respectively. For the ADC(2) method, the MAE is lower by 0.06 eV compared to CIS(D). In contrast, the results are noticeably better in the case of excited states with relatively larger fractions of double excitations. For these transitions, the slightest improvement is 0.07 eV for PBE0-2, while the most significant is 0.16 eV for the SCS RS-DH approach. Concerning the oscillator strengths, we can state that it is difficult to compete with the ADC(2) method; however, significant improvements can be realized in the case of the ADC(2)-based functionals. As it is obvious, the results are well-balanced within the group of the genuine and ADC(2)-based approaches regardless of the exchange and correlation functionals applied or the XC energy expressions used. The lowest MAE of, precisely, 0.027 is attained by ADC(2). The error is noticeably higher for the ADC(2)-based functionals, being around 0.070; however, it is almost three times higher for the genuine DHs compared to the ADC(2)-based approaches. Similar observations can be made if the relative errors are considered, which are below 30% for the present RS DHs, while they are around 80% for the best CIS(D)-based approaches.

**Figure 6:**
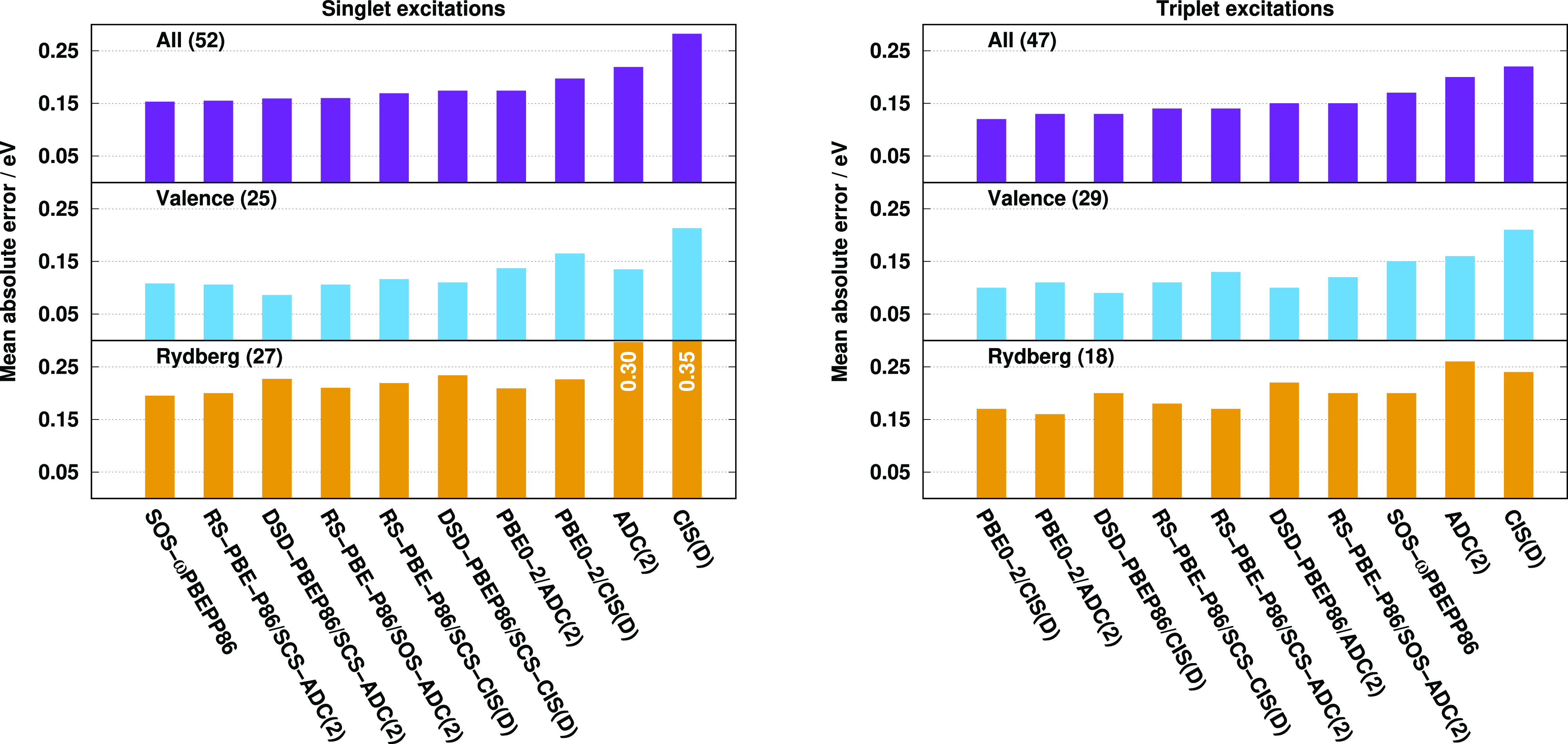
MAEs for calculated singlet (left) and triplet (right) excitation energies for the LJ1 test set^139^ using aug-cc-pVTZ basis sets with corresponding auxiliary bases. The numbers of transitions are in parentheses.

Next, we compare the performances for the LJ1 test set. The results are collected in Figure 6. Inspecting the MAEs for the singlet excitations, we can conclude that the overall errors are well-balanced for almost all the functionals. The superiors are the SOS-ωPBEPP86 and RS-PBE-P86/SCS-ADC(2) approaches with a MAE of 0.15 eV, while the error is under 0.17 eV for the others, except for PBE0-2/CIS(D), where it is 0.19 eV. The inferiors are the wave function-based methods; however, the ADC(2) results are noticeably better. Again, the overall performance of the ADC(2)-based approaches is slightly superior to the genuine DHs. The improvement is 0.02 eV for all the functionals. The valence results are in line with the expectations. Outstanding accuracy can be obtained for the DSD-PBEP86/SCS-ADC(2) method with a MAE of 0.09 eV; however, highly acceptable results are provided by the SOS-ωPBEPP86 and ADC(2)-based RS-DH functionals as well, where the error is only 0.11 eV. The PBE0-2 results are somewhat salient as the MAEs are 0.14 and 0.17 eV for the ADC(2)-based and genuine approaches, respectively. Inspecting the Rydberg excitations, the errors are well-balanced, and salient functionals cannot be identified. The lowest MAEs, 0.20 eV, are attained by the best two performers. The DSD-PBEP86 functionals are inferiors; however, the errors are only 0.23 eV in both cases, which are highly acceptable. In general, the Rydberg errors are higher compared to the valence results, while significant differences cannot be observed between the SCS and SOS ADC(2)-based RS DHs.

Again, the triplet errors are somewhat smaller compared to the singlet ones, but the picture somewhat changes for them. Despite the poor singlet results, the lowest MAEs, 0.12 and 0.13 eV, are attained by the genuine and ADC(2)-based PBE0-2 approaches, respectively. The error starts to increase slightly, but it is still below 0.15 eV for almost all the functionals. The largest overall error is obtained for SOS-ωPBEPP86 with a MAE of 0.17 eV; however, it is still more accurate than the wave function-based methods. Significant differences cannot be observed between the ADC(2)-based and genuine RS DHs, while the latter ansatz provides negligibly better results for the PBE0-2 and DSD-PBEP86 approaches. Inspecting the triplet valence excitations, similar observation can be made as for the singlet ones. The errors are fairly well-balanced. The DSD-PBEP86 results are outstanding, while the PBE0-2 and RS-DH functionals have a similar accuracy with MAEs of around 0.11 eV, which are highly acceptable. For the Rydberg excitations, the lowest error is 0.16 eV achieved by the PBE0-2/ADC(2) approach, while the RS-PBE-P86/SCS-ADC(2) and PBE0-2/CIS(D) methods, where the MAE is only higher by 0.01 eV, are also outstanding. For the remaining functionals, the error hardly exceeds 0.20 eV. Comparing the ADC(2)-based RS DHs, the SCS variant is more suitable for Rydberg excitations, while the valence results are somewhat better for the SOS variant.

#### 3.3.3 Test Set of Loos, Jacquemin, and Co-Workers

The compilation of the further statistical error measures for the LJ1 set can be found in Table 4. The lowest MEs can be achieved through significant error cancellation for the singlet excitations. As it can be seen, the ME has an opposite sign for the valence and Rydberg transitions for the best performers, such as ADC(2) and DSD-PBEP86/SCS-CIS(D). The error is moderate for SOS-ωPBEPP86, where the error cancellation is less significant. We note that the lowest overall SDs and RMSEs were provided by the SOS-ωPBEPP86- and ADC(2)-based RS-DH approaches by far. Interestingly, in the case of the PBE0-2 and RS-DH methods, the valence and Rydberg excitations are systematically overestimated. The lowest maximum error, 0.50 eV, is attained by the SOS-ωPBEPP86 and RS-PBE-P86/SOS-ADC(2) functionals, while it is tolerable for the DSD-PBEP86 and other RS-DH methods. The PBE0-2 approaches are inferiors with a MAX of around 0.76 eV. In general, the MEs and MAXs are higher for the Rydberg states compared to the valence transitions as it was so for the MAEs. For the triplet excitations, at the same time, the lowest ME and the highest RMSE are obtained by SOS-ωPBEPP86. The MEs are highly acceptable for the PBE0-2 and RS-DH functionals, while they are more remarkable for the DSD-PBEP86 approaches. In general, the MEs are somewhat higher for the Rydberg excitations, while the MAXs belong to valence transitions. For both the singlet and the triplet transitions, again, a systematic red-shift can be observed between the ADC(2)-based and the genuine approaches.

**Table 4:** Additional Error Measures for Calculated Excitation Energies (in eV) for the LJ1 Set^139^ Using aug-cc-pVTZ Basis Sets with Corresponding Auxiliary Bases^a^

	Singlet excitations						Triplet excitations					
	All (55)		Valence (26)		Rydberg (29)		All (47)		Valence (29)		Rydberg (18)	
Methods	ME	MAX	ME	MAX	ME	MAX	ME	MAX	ME	MAX	ME	MAX
CIS(D)	0.08	1.03	0.16	0.56	–0.01	1.03	0.12	0.61	0.21	0.61	–0.03	0.53
ADC(2)	–0.03	0.71	0.09	0.50	–0.13	0.71	0.02	0.69	0.12	0.55	–0.13	0.69
SOS-ωPBEPP86	0.04	0.50	–0.01	0.24	0.08	0.50	0.00	0.88	–0.08	0.88	0.13	0.52
RS-PBE-P86/SCS-CIS(D)	0.10	0.63	0.08	0.32	0.12	0.63	0.02	0.72	–0.03	0.72	0.10	0.54
RS-PBE-P86/SCS-ADC(2)	0.07	0.57	0.04	0.28	0.10	0.57	–0.03	0.69	–0.07	0.69	0.05	0.47
RS-PBE-P86/SOS-ADC(2)	0.07	0.50	0.06	0.28	0.09	0.50	0.04	0.79	0.01	0.79	0.10	0.53
DSD-PBEP86/SCS-CIS(D)	–0.03	0.54	0.07	0.25	–0.12	0.54	–0.06	0.48	–0.03	0.48	–0.11	0.45
DSD-PBEP86/SCS-ADC(2)	–0.05	0.54	0.04	0.23	–0.13	0.54	–0.09	0.50	–0.06	0.46	–0.13	0.50
PBE0-2/CIS(D)	0.14	0.77	0.14	0.35	0.14	0.77	0.02	0.73	–0.02	0.73	0.09	0.48
PBE0-2/ADC(2)	0.11	0.76	0.10	0.34	0.12	0.76	–0.01	0.71	–0.05	0.71	0.05	0.43

a The numbers of transitions are in parentheses.

**Figure 7:**
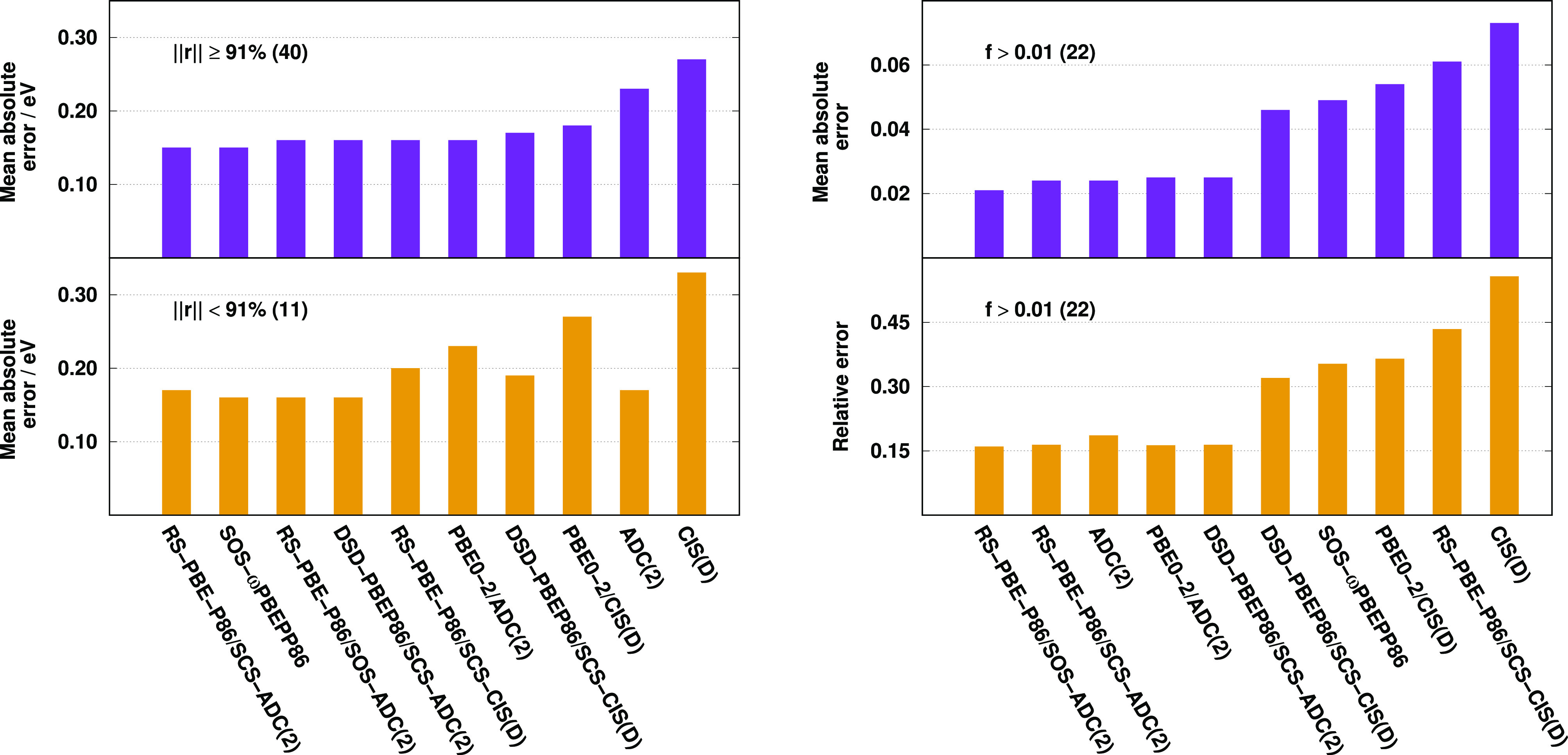
Error measures for calculated singlet excitation energies with different fractions of single excitation coefficients (left) and oscillator strengths (right) for the LJ1 test set.^139^ The numbers of transitions are in parentheses.

As the weights of the single excitations in the CC3 wave function and oscillator strengths are also available for the LJ1 test set, the same comparisons were carried out as for the Thiel set. In this case, transitions where ∥**r**∥ ≥ 91% are considered as a singles dominated excitation, while the remainders are treated as states with larger fractions of double excitations. The results are visualized in Figure 7. Considering the DH functionals, again, the results are fairly well-balanced for the singles dominated excitations. The ADC(2)-based approaches outperform the CIS(D)-based counterpart in all the cases; however, the difference is negligible. The improvement is only 0.02 eV for the PBE0-2 approach, which is the most notable case. For the ADC(2) method, the MAE is lower by 0.04 eV compared to CIS(D). For the transitions with relatively larger weights of double excitations, these gains are more remarkable as the difference is 0.03 eV for the DSD-PBEP86 and SCS RS-DH functionals, while it is 0.04 eV for PBE0-2. The good performance of the CIS(D)-based SOS-ωPBEPP86 approach is surprising. For the oscillator strengths, the best performances are attained by the RS DH-ADC(2) approaches. For both measures, the present approaches are even better than the ADC(2) method, while the remaining ADC(2)-based functionals significantly outperform the genuine counterpart. Using the ADC(2)-based ansatz, the relative error fluctuates around 15%, while it is at least twice as large for the CIS(D)-based functionals.

#### 3.3.4 Intermolecular CT Set

**Figure 8:**
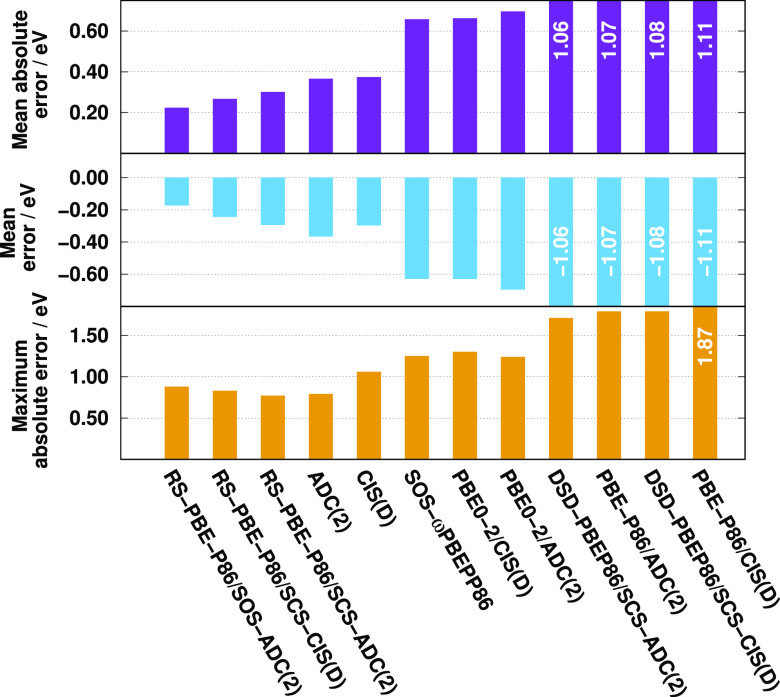
Error measures for calculated singlet excitation energies for the intermolecular CT test set^141^ using cc-pVDZ basis sets with corresponding auxiliary bases.

Finally, we study intermolecular CT excitations, which present a well-known problem even for this class of methods.^96,113,114^ This comparison also includes the PBE-P86/CIS(D) and PBE-P86/ADC(2) functionals obtained in Section 3.2. These approaches provided outstanding accuracy for the Gordon training set; however, as we will see it, they are inferiors for CT excitations. The numerical results for the CT benchmark set of Szalay et al.^141^ are presented in Figure 8. As it can be seen, the RS-DH methods are by far superior to the other ones. The lowest MAE, 0.22 eV, is attained by RS-PBE-P86/SOS-ADC(2). The error is still below 0.30 eV for the SCS variant, while its CIS(D)-based counterpart is a bit more accurate. These functionals provide better results than the wave function-based methods, where the MAE is around 0.37 eV. Surprisingly, despite the long-range correction, the SOS-ωPBEPP86 method is not reliable as its error amounts to 0.66 eV. To be fair, we mention that other LC DHs from the Goerigk group provide satisfying results for the same test set;^90^ however, as can be seen, not all LC DHs are suitable for challenging intermolecular CT excitations. In this regard, the ωB2GPPLYP approach^95^ is also reliable with a MAE of 0.39 eV; however, as it was pointed out in ref (98), its performance for general applications is far from the best LC-DHs. The standard DHs are also highly not recommended. The MAE is barely tolerable, 0.66 eV, for the PBE0-2 functionals but is around 1.00 eV for the DSD-PBEP86 and even worse for the PBE-P86 approaches. The excitation energies are systematically underestimated for all the approaches. In the case of the best performers, the ME is somewhat smaller than the corresponding MAE, while these two values are practically identical for the others. The lowest MAXs are produced by the SCS RS-DH methods, while it is around 1.30 eV for the SOS-ωPBEPP86 and PBE0-2 functionals. This relatively large number is still tolerable since the maximum error is at least 1.70 eV for the inferiors. For such excitations, significant differences cannot be observed between the ADC(2)-based and the genuine approaches.

### 3.4 Overall Performance for Simple Cases

**Figure 9:**
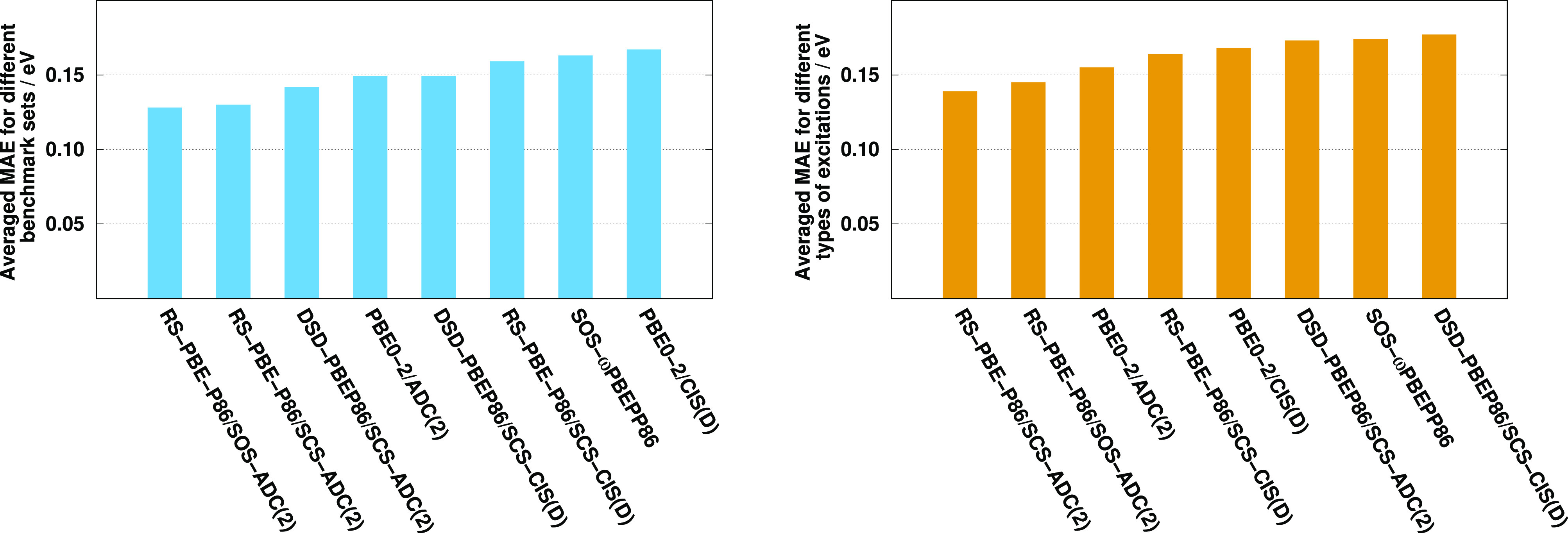
Averaged MAE for different benchmark sets (left) and different characters of excitations (right). Intermolecular CT results are excluded in this comparison. See text for explanation.

It is hard to characterize the performance of the functionals with a single measure. A procedure was recently proposed by Casanova-Páez and Goerigk^98^ where the MAEs were averaged for all the benchmark sets assessed. We use the same measure in this study; furthermore, an additional measure is also introduced where the MAEs are averaged for different characters of excitations. Thus, in the case of the first descriptor, we divide the Gordon, Thiel, and LJ1 benchmark sets into singlet and triplet subsets of excitations, and the resulting six MAEs are averaged. In addition, for the second measure, all the valence and Rydberg transitions regardless of the benchmark sets are split up into singlet and triplet subsets as well, and the four MAEs obtained are averaged. To be fair, the challenging CT benchmark set is omitted in this comparison as those values would bias our results. Accordingly, this ranking is relevant for simple and general applications if only the excitation energies are required. The results are visualized in Figure 9. Inspecting the bars, a couple of important observations can be made. First, the most accurate and robust results are attained by the ADC(2)-based RS DHs. Second, the overall performance of the ADC(2)-based approaches is always better compared to the genuine counterparts. We would like to emphasize that the main differences between the two approaches have less influence on these results. The most significant gains can be achieved for the oscillator strengths and transitions with larger fractions of double excitations. Finally, the PBE0-2 and DSD-PBEP86 functionals are only suggested for certain types of excitations, while the overall performance of SOS-ωPBEPP86 is not consistently better than either standard DHs for the benchmark sets studied, and its failure for challenging intermolecular CT excitations was pointed out.

**Figure 10:**
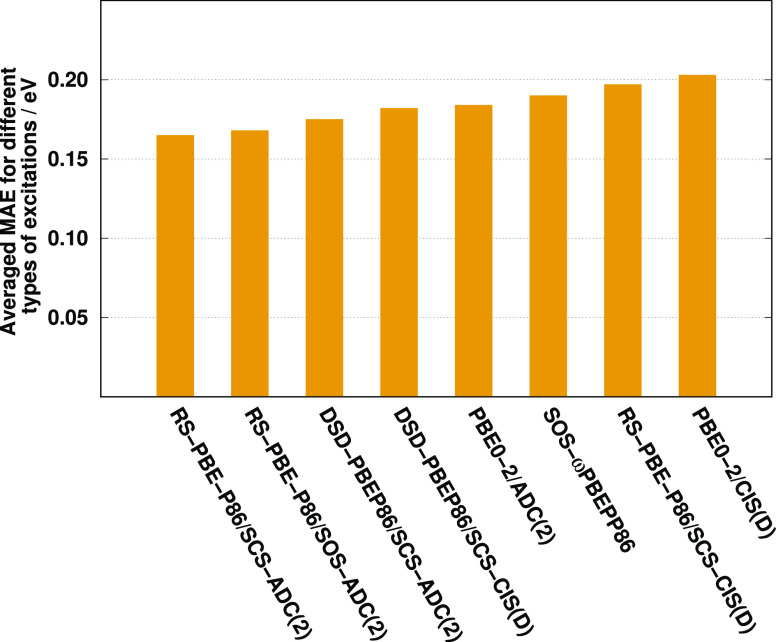
Averaged MAEs for singlet and triplet valence and Rydberg excitations for the combined benchmark set. See text for explanation.

To eliminate the bias caused by the overlapping test sets and to minimize the influence of our training set, an additional comparison was also carried out. In this case, the duplicates were completely excluded; that is, only unique molecules were selected from the benchmark sets. For this purpose, we retained the entire LJ1 test set as the most comprehensive benchmark set used in this study. This set was supplemented with the molecules from the Thiel set that are not included in LJ1. In addition, the molecules from the Gordon test set that were not part of the joint set were also added to the compilation. This results in 169 singlet (140 valence and 29 Rydberg) and 114 triplet (96 valence and 18 Rydberg) excitations for 41 molecules. Here 3 (11), 20 (173), and 18 (99) molecules (excitations) were selected from the Gordon, Thiel, and LJ1 test sets, respectively. Thereafter, similar to the previous paragraph, all the valence and Rydberg transitions regardless of the benchmark sets were split up into singlet and triplet subsets, and the four MAEs obtained were averaged. The results are visualized in Figure 10.

As it can be seen, again, the best performers are the spin-scaled ADC(2)-based RS-DH approaches, and the ADC(2)-based functionals always outperform the genuine counterparts. In contrast to the previous results, the overall performances of the DSD-PBEP86 functionals are better compared to PBE0-2. Since the fitting set does not influence the PBE0-2 and DSD-PBEP86 results, this suggests that the Thiel set is somewhat overweighted in this scheme. The less favorable performance of RS-PBE-P86/SCS-CIS(D) also supports this finding as, similar to PBE0-2, it is inferior for the above-mentioned test set.

## 4 Conclusions

Our ADC(2)-based DH ansatz^80^ has been combined with range-separation techniques. This scheme can be considered as the extension of the robust RS-DH approach relying on the CIS(D)-based ansatz, where both the exchange and correlation contributions are range-separated.^89^ In the new methods, the double excitations are treated iteratively, while the transition moments are evaluated at a higher level taking into account the effect of second-order contributions. To obtain more efficient approaches, spin-scaling techniques were also applied.^90^ The proposed approaches contain three and four empirical parameters in the case of the SOS and SCS variants, respectively. These mixing factors were determined using the well-balanced benchmark set of Gordon et al.;^135^ thereafter, a cross-validation was performed on several popular benchmark sets. In total, 250 singlet and 156 triplet excitations were involved in this study; furthermore, 80 oscillator strengths were also assessed. On top of this, concerning the overall performances, an additional comparison was also carried out where only the unique molecules are considered.

Our numerical results show that the range separation for the correlation contributions is highly recommended for both the ADC(2)- and CIS(D)-based schemes. In addition, the ADC(2)-based approaches slightly but consistently outperform the corresponding genuine counterparts for simple cases, while significant gains can be realized for the oscillator strengths and transitions with larger fractions of double excitations. Ranking the functionals, the most accurate and robust results were attained by the present RS-PBE-P86/SCS-ADC(2) approach and its SOS variant. Significant differences cannot be observed between them; perhaps the SCS variant is a bit more suitable for Rydberg excitations, while the valence results are somewhat better for the SOS variant. In both cases, the averaged MAE is below 0.15 eV, while the relative error of the oscillator strengths is around 25%. Accordingly, the fourth-order scaling RS-PBE-P86/SOS-ADC(2) is highly recommended for general applications. The overall performance of the recently proposed LC SOS-ωPBEPP86 approach^98^ is not consistently better than either the PBE0-2/CIS(D) or the DSD-PBEP86/CIS(D) functionals for the benchmark sets studied. For the oscillator strengths, within the CIS(D)-based ansatz, the lowest relative error is 66% obtained by DSD-PBEP86/SCS-CIS(D). Thus, the error can be reduced by around 65% using the ADC(2)-based ansatz. In addition, for the challenging intermolecular CT excitations, among the density functional approximations assessed in this study, only the RS-DH functionals provided reliable results.

## Supporting Information Available

Computed excitation energies and oscillator strengths (XLSX)
